# Caudal regression syndrome without maternal diabetes mellitus

**DOI:** 10.1136/bcr-2022-253136

**Published:** 2023-03-23

**Authors:** Naomi Mwamanenge, Haika Mariki, Martha Mkony, Karim Premji Manji

**Affiliations:** 1 Department of Paediatrics and Child Health, Muhimbili University of Health and Allied Sciences, Dar-es-Salaam, Tanzania, United Republic of; 2 Pediatrics, Muhimbili National Hospital, Dar es Salaam, Tanzania, United Republic of; 3 Department of Paediatrics and Child Health, Muhimbili National Hospital, Dar es Salaam, Tanzania, United Republic of

**Keywords:** Disability, Congenital disorders, Paediatrics

## Abstract

Caudal regression is a rare complex disorder impacting the formation of the caudal segment of the spine and spinal cord. We report a preterm newborn baby who was referred to us due to respiratory distress syndrome and bilateral knee contracture. A clinical examination and a radiographic skeletal survey revealed a short spinal cord with complete agenesis of the lumbar, sacrum and coccygeal spine, and hypoplastic iliac bones with bilateral knee contractures. The mother did not have diabetes. The long-term outcome is not well-known in our set-up.

## Background

Caudal regression syndrome (CRS) is a rare complex disorder impacting the formation of the caudal segment of the spine and spinal cord. The anomalies of the distal spinal segment can range from sacral agenesis, genital malformation, cardiac anomalies, pulmonary hypoplasia or aplasia, renal dysplasia or aplasia, anomalies of the lower extremities including arthrogryposis and anorectal malformation.^1^ Maternal diabetes is a major risk factor for developing CRS presenting as sacral agenesis syndrome and hypoplasia of the lower extremities.^2 3^ This defect is thought to occur during the fourth to seventh week of embryogenesis as a result of impaired development of the mesoderm.^3^

## Case presentation

A term male neonate was referred to our tertiary hospital from one of the primary hospitals in the city for respiratory support and orthopaedic management. He was delivered via assisted breech delivery at 30 weeks’ gestation with an APGAR score of 7 and 10 at the first and fifth, minutes, respectively, with a birth weight of 1.9 kg.

Both parents are in their early 30s and with no comorbidities. This is their third child, and the other two siblings are well. There was no family history of similar condition in the parents or the two older siblings. The parents are non-consanguineous. The mother started to attend antenatal clinics in the second trimester. She was not given folic acid however was given malaria prophylaxis at 20 weeks’ gestation that comprised of three tablets stat of sulfadoxine/pyrimethamine (1500/75 mg) and an iron supplement of 60 mg tablets of ferrous sulfate daily. This malaria prophylaxis was given on two occasions only at 20 weeks and 24 weeks. She had two antenatal ultrasound scans, both reported normal findings. She was normotensive throughout the pregnancy and had no history of gestational diabetes with negative serology results for syphilis using the VDRL and HIV infection. Her random blood glucose at 20 weeks was 5.5 mmol/L and a repeat at 28 weeks was 5.4 mmol/L; she was euglycaemic and the attending obstetrician did not do the HBA1C. This was done 2 months after delivery and it was 5.1%, which is normal. There is no history of diabetes or similar genetic illness in the family. There is no exposure to any other drugs like minoxidil, sodium valproate or creams with retinoic acid.

**Figure 1:**
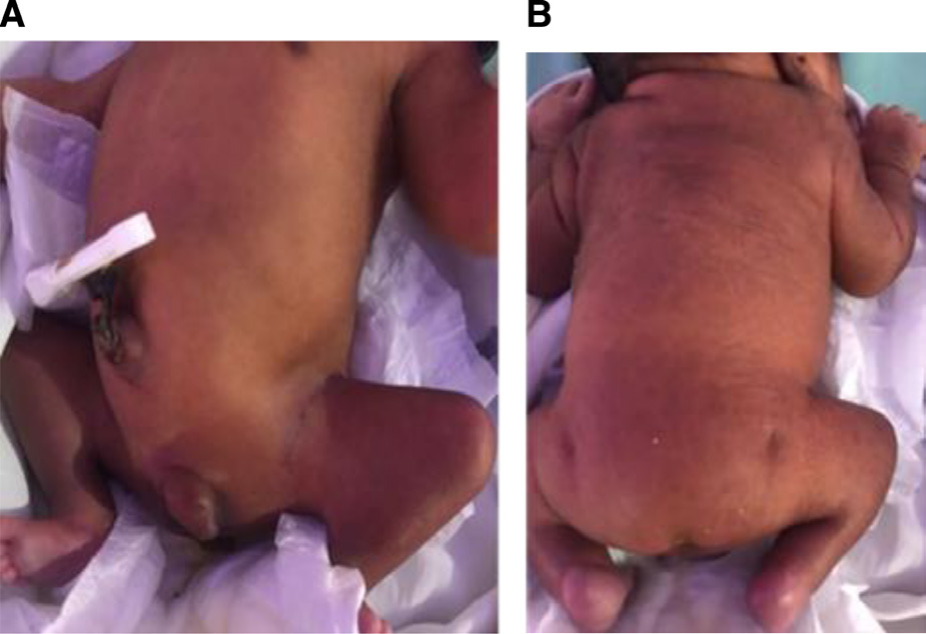
(A) (Flexed hip joints with flexion contractures of both knees (’frog-like posture’). (B) Caudal regression syndrome with bilateral deep dimples at the femoral trochanter area.

On admission, the baby had moderate respiratory distress with Silverman-Andersen score of 4 with central cyanosis. The respiratory rate was 70 breaths/min, oxygen saturation was 70% and heart rate was 147 beats/min. There was a pan-systolic murmur, and 100% oxygen supplementation did not seem to improve cyanosis. On examination, he had a short neck and low-set ears, otherwise no obvious facial deformity. Both lower limbs appeared short with flexion knee and hip contracture putting the baby in splayed posture; upper limbs were normal. He had bilateral undescended testes and microphallus of 1 cm and prominence of the spine at T12 vertebra; his lumbar and sacral bones could not be palpated and he had bilateral deep dimples at the femoral trochanter area (figure 1A). Sensation was present in both lower limbs. He has patulous anus with faecal incontinence. The occipital frontal circumference was 28 cm and length was 40 cm.

The baby required assisted ventilation using the continuous positive airway pressure, at low settings (pressure of 5–6 and fractional inspired oxygen of 30%–40%). He was weaned off oxygen on day 3 of life, his saturation ranging 90%–93%. He was initiated on expressed breast milk via an orogastric tube and later transitioned to intermittent direct breast feeding.

## Investigations

**Figure 2:**
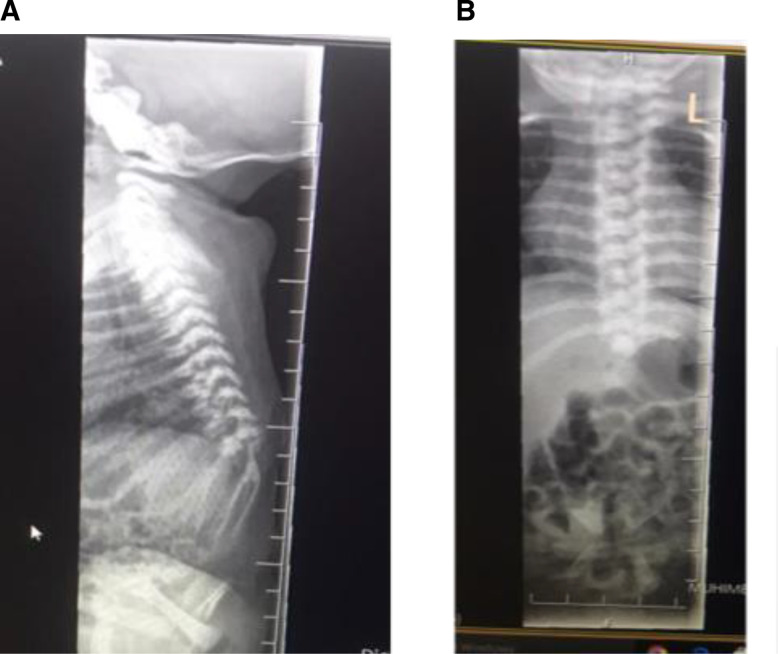
(A) Skeletal survey showing short spinal cord with complete agenesis of the lumbar, sacrum and coccygeal spine. (B) Skeletal survey showing absence of iliac bone, sacrum and hip joints.

A full skeletal survey, echocardiogram and abdominal ultrasound were done. The skeletal survey revealed short spinal cord with complete agenesis of the lumbar, sacrum and coccygeal spine, and hypoplastic iliac bones (figure 2A, B).

Abdominal ultrasound did not show any visceral malformation; both kidneys were of normal size and location, and the liver and spleen were also normal. Pelvic ultrasound revealed an absence of sacrum.

**Figure 3:**
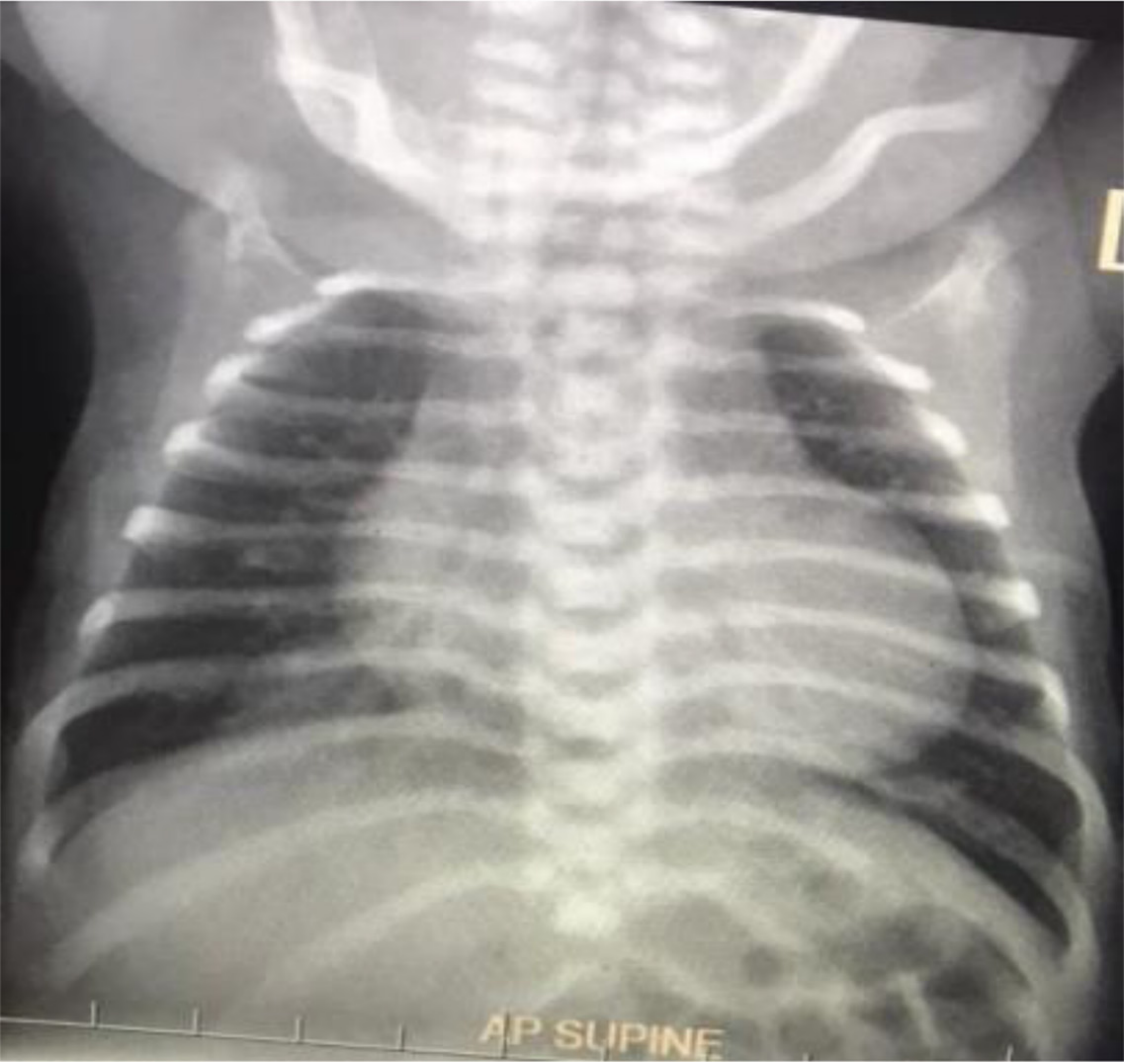
Chest X-ray showing cardiomegaly with ‘egg-on-a-string’ shape feature suggestive of transposition of great arteries.

Chest X-ray showed cardiomegaly with ‘egg-on-a-string’ shape feature suggestive of transposition of great arteries. There was cardiomegaly and oligaemic lung shadow (figure 3).

Echocardiogram revealed situs solitus, pulmonary artery arising from left ventricle, mild pulmonary stenosis, aorta arising from right ventricle, large ventricular septal defect (VSD) size 6.7 mm with bidirectional flow, dextro-transposition of great arteries (DTGA) with large VSD, moderate ostium secundum atrial septal defect and mild pulmonary stenosis.

## Differential diagnosis

The differential diagnosis is Currarino syndrome, which presents with a triad of sacral agenesis, anal atresia/stenosis and presacral mass. Our case did not have a sacral mass.

## Treatment

Treatment is mainly supportive. Our patient was reviewed by a cardiologist and scheduled for a monthly follow-up while waiting for the correction of DTGA. He was also reviewed by an orthopaedic surgeon where correction of the contracture will be done once cardiac condition is corrected. The agenesis of the sacrum cannot be remedied, but physiotherapy and occupational therapy will be needed intensively as he grows.

## Outcome and follow-up

The baby is having regular monthly follow-up clinics with the cardiologist and orthopaedic surgeons.

## Discussion

CRS is very rare and occurs in approximately 1:100 000 live births.^1 2^ Th embryogenesis at 4–7 weeks of life is characterised by failure of development of the lumbosacral vertebra and caudal spinal cord due to defects in the development of the caudal mesoderm and the structures that it ultimately forms.^3^

The teratogenic insult caused by hyperglycaemia has been found to be one of the major factors for CRS. Hyperglycaemia increases the risk of malformation to the fetus by negatively impacting the DNA structure. The role of genetic mutation of the VANGL1 gene, which is inherited as an autosomal dominant trait located in short arm (p) chromosome 1 (1p13), has been found.^4^ Unfortunately, in our country, genetic studies are not done; however, samples can be sent abroad. This is usually very expensive and results take almost 8 weeks–4 months to be reported.^5 6^

The mother of the patient we present did not have any risk factors during antenatal period. She was normotensive and had no diabetes mellitus during pregnancy or otherwise. Some studies have shown an association between folic acid and CRS. Environmental factors also play a role in the development of caudal regression. These include hypoxia, retinoic acid, radiation and alcohol consumption. When retinoic acid homeostasis is impaired during the first trimester, it affects both embryogenesis and organogenesis/agenesis through impaired cell migration and differentiation.^6 7^

The detrimental and irreversible effects of folate deficiency on the fetus are well established.^8^ An inadequate concentration of periconceptional supplementation of folic acid impairs/inhibits DNA synthesis and other cellular processes.^9^ Fetal structural anomalies may also be prevented by folic acid by regulation of homocysteine metabolism especially when given in the first trimester.^7 10^ Antenatal supplementation of folic acid is crucial; however, our patient started antenatal clinics in the second trimester and therefore was not given folic acid.^7 8^ The antenatal ultrasound done in the second trimester could not pick any anomaly and calls for a proper expert ultrasonography with the intent to visualise anomalies.

CRS is rare, and its early recognition is important. It can be detected prenatally and just like other such highly lethal malformations, interventions can be instituted early. Its prevalence in infants of mothers with diabetes is reported at 1 in 350 live births, which includes all the variants.^11^

The CRS is 250 times more common among offspring of mothers with diabetes. The absence of diabetes should prompt doctors to look into other possible causes, such as maternal exposure to cocaine or alcohol consumption, vascular steal or hypoperfusion, fetal hypoxaemia and amino acid imbalance.^11 12^

Family history and maternal diabetes mellitus are two of the risk factors for this disorder. Of all the CRS, 16% are related to maternal diabetes (the abnormal embryological development of the caudal mesoderm occurs within the first 4 weeks of embryonic development).^13^

In our case, none of the possible risk factors were noted.

This case is reported to highlight the early diagnosis of the syndrome, and the difficulties in diagnosis and treatment postnatally. It is indeed a rare condition and can be associated with various abnormalities and poor quality of life and increased mortality, so recognition early on during antenatal period is important.

Classification of CRS done by Renshaw in 1978 describes the types of defect and articulation between bones.^6^ According to this classification, our patient had type III CRS.

Management is a multidisciplinary approach aimed at supportive care in order to improve functionality of the deformity and to reach their highest potential. Our patient has been reviewed by the cardiologist and orthopaedic surgeon, who are following the patient closely. The orthopaedic surgeon will correct the contracture and further assist the baby with orthopaedic support in order to maintain functionality once the cardiac anomaly has been resolved.

Prognosis depends on the degree of the defect in the vertebra and spinal cord and other associated malformations including bowel or urinary bladder incontinence.^9^ Our patient, in addition to orthopaedic deficit, has faecal and urinary incontinence. No cognitive impairment has been reported. Patients with CRS lack motor skills below the level of the normal spine; however, sensation is intact just like in our patient, and it could be explained due to fairly normal segmental development of the neural tube at the level of agenesis.

Long-term follow-up is required for growth and developmental milestone including orthopaedic reviews for improving pelvic stability and hence functionality and nephrologist assessment in view of neurogenic bladder that may develop later on in life.

**Patient’s perspective**

I am sad because my baby has a skeletal deficit even though the antenatal ultrasound that I did were reported normal. I am also sad because I was told that my baby has a cardiac problem even though the cardiologist will correct the problem next month and orthopedic doctors will follow up with my baby to help with the knee problem.

**Learning points**

- The novel information is that this is a rare condition, but information is scarce especially from Africa.
- Early diagnosis during antenatal period is possible if there is an index of suspicion from a family history. Now that this case has been reported, there is going to be increased awareness in our setting.
- As for this case being sporadic or possibly genetic, we do not have genetic testing, and all other possible conditions predisposing to caudal regression syndrome were not present, such as diabetes, hypertension, substance abuse, smoking, alcohol and so on.
